
JOURNAL INFORMATION
==============================
NLM Title Abbreviation: ScientificWorldJournal
Journal ID: ScientificWorldJournal
NLM Journal ID: 101131163
Journal ID: TSWJ

The Scientific World Journal

ISSN: 2356-6140
EISSN: 1537-744X
Publisher: Wiley

ARTICLE INFORMATION
==============================
PMCID: PMC6083918
PMID: 30147478
DOI: 10.1100/tsw.2001.163
Article ID: 289705
Article version: 1
Subjects: Short Report

Coordinated Activation of Caspases and Apoptosis in Allogeneic Antigen Stimulated Human T Cells

Jayaraman Sundararajan 1 *
Barrett John N. 2
1 Department of Pathology, University of Miami School of Medicine, 1450 NW 10th Avenue, DRI Building Room 5018, Miami, FL 33136, USA
2 Department of Physiology, University of Miami School of Medicine, 1450 NW 10th Avenue, DRI Building Room 5018, Miami, FL 33136, USA
*Sundararajan Jayaraman: sjayaram@med.miami.edu
Publication date: 2001
Electronic publication date: 2001 Jan 12
Volume: 1
First page: 37
Last page: 37
Copyright: Copyright © 2001 Sundararajan Jayaraman and John N. Barrett.
Copyright year: 2001
License: This is an open access article distributed under the Creative Commons Attribution License, which permits unrestricted use, distribution, and reproduction in any medium, provided the original work is properly cited.
License URL: https://creativecommons.org/licenses/by/3.0/

==============================
Miami *Nature Biotechnology* Short Reports
*TheScientificWorld* (2001) 1(S3), 37SR
ISSN 1532-2246; DOI 10.1100/tsw.2001.163

### COORDINATED ACTIVATION OF CASPASES AND APOPTOSIS IN ALLOGENEIC ANTIGEN STIMULATED HUMAN T CELLS

Sundararajan Jayaraman[1,*] and John N. Barrett[2]

[1]Department of Pathology and [2]Department of Physiology, University of Miami School of Medicine, 1450 NW 10th Avenue, DRI Building Room 5018, Miami, FL 33136, USA
* Sjayaram@med.miami.edu

**INTRODUCTION.** Activation of caspases is generally associated with induction of apoptosis in many cell types (1). Stimulation of normal human T lymphocytes with allogeneic antigens or mitogen leads to cell proliferation as well as caspase-3 activation, suggesting that caspases may also have functions other than apoptosis (2-3). However, the role of caspases in human T cell apoptosis remains unclear. The aim of our study was to define the role of caspases in human T cell apoptosis.

**METHODS.** Normal peripheral blood T lymphocytes were stimulated *in vitro* with γ-irradiated allogeneic peripheral blood mononuclear cells and IL-2. Intact cells were assessed by flow cytometry for propidium iodide uptake and phosphatidylserine (PS) externalization using fluorochrome conjugated annexin V. Intact cells were also analyzed by flow cytometry as well as confocal microscopy for the simultaneous binding of annexin V and FMK-VAD-FITC, a cell permeable caspase inhibitor that binds to activated caspases. Immunoblotting and enzymatic assays were respectively performed in whole cell lysates to determine the proteolytic cleavage of caspases and the ability of activated caspases to cleave fluorogenic substrates *in vitro*.

**RESULTS.** Freshly isolated normal peripheral blood mononuclear cells did not contain apoptotic cells or processed caspase-3. After 5 to 8 days of allogeneic antigen stimulation, 30 to 40% of T cells became apoptotic as determined by a combination of propidium iodide uptake and annexin V binding. A good correlation between annexin V binding and *in situ* detection of activated caspases using FMK-VAD-FITC was found in this population. Western blotting of whole cell lysates showed the proteolytic cleavage of caspase-3 into 22-kDa and 17-kDa subunits in cultured T cells. This was accompanied by detectable levels of *in vitro* cleavage activity against the caspase-3 substrate, DEVD-AMC. Confocal microscopy showed externalization of PS in all of the T cells with caspase activation detected by FMK-VAD-FITC labeling. Activation with an agonist anti-Fas antibody increased the level of apoptosis, binding of FMK-VAD-FITC, caspase-3 enzymatic activity and the cleavage of caspase-3 into a smaller 12-kDa subunit. Importantly, the broad-spectrum cell permeable caspase inhibitor zVAD (OMe)-FMK blocked anti-Fas antibody-induced apoptosis, activation of intracellular caspases, and the cleavage of 22-kDa caspase-3 into smaller subunits.

**DISCUSSION.** Our data demonstrate that *in vitro* activation of normal human T cells with allogeneic antigens leads to apoptosis in a significant proportion of T cells as indicated by permeability to propidium iodide and PS externalization. Caspase-3, a key enzyme required for apoptosis in several model systems (1) is activated by proteolytic cleavage in cultured T cells. Confocal microscopy showed externalization of PS in all of the cells with caspase activation detected by FMK-VAD-FITC labeling. This suggests a linkage between caspase activation and apoptosis in T cells. zVAD (OMe)-FMK inhibition of both Fas antibody-induced caspase-3 activation and apoptotic cell death indicates that caspases have a role in human T cell apoptosis. Thus, our data suggest that in addition to a role in T cell activation (2-3), caspases, particularly caspase-3 may have a critical role in mediating apoptotic death of antigen stimulated normal human T lymphocytes.

**REFERENCES.**

1. Nicholson, D.W. (1999) Cell Death Differ. 6, 1028-1042
2. Miossec, C., Dutilleul, V., Fassy, F., and Diu-Hercend, A. (1997) J. Biol. Chem. 272, 13459-13462
3. Alam, A., Cohen, L.Y., Aouad, S., and Sekaly, R.-P. (1999) J. Exp. Med. 190, 1879-1890
